# Uremic toxin indoxyl sulfate promotes proinflammatory macrophage activation by regulation of β-catenin and YAP pathways

**DOI:** 10.1007/s10735-020-09936-y

**Published:** 2021-01-02

**Authors:** Ying Li, Jing Yan, Minjia Wang, Jing Lv, Fei Yan, Jin Chen

**Affiliations:** 1 Department of General Practice, Zhejiang Hospital, 12 Lingyin Road, West Lake District, Hangzhou, 310013 Zhejiang People’s Republic of China; 2 Department of Critical Care Medicine, Zhejiang Hospital, Hangzhou, 310013 Zhejiang People’s Republic of China

**Keywords:** Chronic kidney disease, Cardiovascular disease, Indoxyl sulfate, Macrophage polarization

## Abstract

Evidence has been shown that indoxyl sulfate (IS) could impair kidney and cardiac functions. Moreover, macrophage polarization played important roles in chronic kidney disease and cardiovascular disease. IS acts as a nephron-vascular toxin, whereas its effect on macrophage polarization during inflammation is still not fully elucidated. In this study, we aimed to investigate the effect of IS on macrophage polarization during lipopolysaccharide (LPS) challenge. THP-1 monocytes were incubated with phorbol 12-myristate-13-acetate (PMA) to differentiate into macrophages, and then incubated with LPS and IS for 24 h. ELISA was used to detect the levels of TNFα, IL-6, IL-1β in THP-1-derived macrophages. Western blot assay was used to detect the levels of arginase1 and iNOS in THP-1-derived macrophages. Percentages of HLA-DR-positive cells (M1 macrophages) and CD206-positive cells (M2 macrophages) were detected by flow cytometry. IS markedly increased the production of the pro-inflammatory factors TNFα, IL-6, IL-1β in LPS-stimulated THP-1-derived macrophages. In addition, IS induced M1 macrophage polarization in response to LPS, as evidenced by the increased expression of iNOS and the increased proportion of HLA-DR+ macrophages. Moreover, IS downregulated the level of β-catenin, and upregulated the level of YAP in LPS-stimulated macrophages. Activating β-catenin signaling or inhibiting YAP signaling suppressed the IS-induced inflammatory response in LPS-stimulated macrophages by inhibiting M1 polarization. IS induced M1 macrophage polarization in LPS-stimulated macrophages via inhibiting β-catenin and activating YAP signaling. In addition, this study provided evidences that activation of β-catenin or inhibition of YAP could alleviate IS-induced inflammatory response in LPS-stimulated macrophages. This finding may contribute to the understanding of immune dysfunction observed in chronic kidney disease and cardiovascular disease.

## Introduction

Chronic kidney disease (CKD) is defined as functional abnormalities of the kidney, or decreased glomerular filtration rate (GFR, < 60 mL/min/1.73 m^2^) for more than 3 months (Chala et al. 2019; Shiba and Shimokawa 2011). In addition, cardiovascular disease (CVD) is a group of problems of the heart or blood vessels, and is a serious complication of CKD (Weiner 2009). CKD is a serious risk factor for CVD, indicating that kidney disease and CVD are closely interconnected (Yang et al. 2010). Kidney damage cause dysfunction of heart tissue, eventually leading to dysfunction of both organs (Liu et al. 2014).

Previous study indicated that CKD is commonly associated with the inflammation (Engel et al. 2019), and macrophages are the main contributors to the inflammatory response to CKD (Guiteras et al. 2016). In addition, macrophages are divided into 2 groups including M1 (classically activated macrophages) and M2 (alternatively activated macrophages) (Zhou et al. 2019b). M1 macrophages primary exerted a pro-inflammatory role, while M2 macrophages mainly exhibited the anti-inflammatory role (Mosser and Edwards 2008). Moreover, macrophage polarization plays a vital role in the progression of CKD (Engel and Chade 2019). In the early stage, renal injury activated the inflammation response pathway, and promoted M1 macrophage polarization. However, at the later stage, a number of anti-inflammatory cytokines stimulated the production of M2 macrophages, which contribute to kidney repair (Engel and Chade 2019).

Indoxyl sulfate (IS) is an important uremic solute, which normally excreted into urine (Yang et al. 2015). Previous study indicated that the level of IS was significantly increased in patients with CKD (Adijiang et al. 2011). Decreased GFR in patients with CKD could result in a reduction in IS excretion, and then the concentration of IS was gradually accumulated in uremic serum (Niwa et al. 1988). In that situation, IS could accelerate the progression of CKD (Miyazaki et al. 1997). In addition, IS has been shown to be involved in the development of CVD (Watanabe et al. 2019). Tan et al. indicated that IS could induced cardiomyocyte toxicity (Tan et al. 2018). Evidence has been shown that IS could affect kidney and cardiac functions (Lekawanvijit et al. 2010). However, the role of IS in macrophage polarization in LPS-induced inflammatory conditions remain unclear. Thus, in this study, we aimed to investigate the effect of IS on macrophage polarization during LPS challenge.

## Materials and methods

### Cell culture

Human acute monocytic leukaemia cell line THP-1 was purchased from American Type Culture Collection (ATCC, Rockville, MD, USA). Cells were incubated in RPMI 1640 medium, supplemented with 10% heat-inactivated fetal bovine serum (FBS, Thermo Fisher Scientific, Waltham, MA, USA) and antibiotic–antimycotic solution (100 U/ml penicillin and 0.1 mg/ml streptomycin, Thermo Fisher Scientific) at 37 °C in a humidified atmosphere containing 5% CO_2_. Phorbol 12-myristate-13-acetate (PMA, Sigma Aldrich, St. Louis, MO, USA) was used to induce the differentiation of THP-1 monocytes into macrophages (M0 macrophages). Macrophages were then incubated with 10 μg/mL of LPS in order to obtain M1 macrophages (Genin et al. 2015).

#### ELISA

THP-1 cells were exposed to PMA (160 nM) for 48 h, and then incubated in PMA-free medium for 24 h, following by different concentrations of LPS (0, 10 or 100 μg/mL) and IS (0, 0.25, 0.5, 1 or 2) for 24 or 48 h. The concentrations of TNFα, IL-6, IL-1β in the supernatant of macrophages were determined by ELISA (ExCellBIO, Shanghai China) according to the manufacturer's procedures.

### Western blot

The protein concentration was detected using the BCA protein assay kit (Thermo Fisher Scientific). Protein samples (20 μg) were separated on a 10% sodium dodecyl sulfate‐polyacrylamide (SDS-PAGE) gels, and then electro-transferred onto polyvinylidene fluoride (PVDF, Millipore, Billerica, MA, USA) membranes. After that, membranes were blocked with 5% skim milk in TBST for 1 h at room temperature, and then incubated overnight at 4 °C with the following antibodies: Arginase1 (1:1000, Abcam Cambridge, MA, USA), iNOS (1:1000, Abcam), GAPDH (1:1000, Abcam). Later on, the membranes were incubated with the corresponding secondary antibodies (1:5000, Abcam) at room temperature for 2 h. The ECL detection kit (Thermo Fisher Scientific) was used to analyze the protein bands.

### Flow cytometry

Cells were incubated with anti-HLA-DR (M1, macrophage cell subpopulation marker, Abcam) or anti-CD206 (M2, macrophage cell subpopulation marker, Abcam) for 20 min at 4 °C according to the manufacturer's procedures. After washing twice with PBS, cells were resuspended in fluorescence-activated cell sorting (FACS) buffer. Then, fluorescence activated cell sorting was performed by using a FACSAria II instrument (BD Biosciences, Franklin Lake, NJ, USA), and the data were analyzed using FACSDiva 6.1.1 software (BD).

### Immunofluorescence assay

Cells were fixed in 4% paraformaldehyde, and then permeabilized with 0.1% Triton X-100 for 20 min. After that, the cells were blocked with 10% goat serum at room temperature for 1 h. Later on, the cells were incubated with primary antibodies anti-β-catenin (1:1000, Abcam) and Yes-associated protein (YAP, 1:1000, ProteinTECH group Inc., Chicago, Illinois, USA) overnight at 4 °C. Subsequently, the specimens were stained with Goat Anti-Rabbit IgG H&L secondary antibody (Cy3) (1:100, Boster biological Technology Co. Ltd, Pleasanton, CA, USA) on a second day for 2 h at room temperature. Cell nuclei were counterstained with DAPI for 5 min, and then cells were imaged with a laser scanning confocal microscope (LSM, Carl Zeiss).

### Statistical analysis

GraphPad Prism 7 (GraphPad Software, Inc., La Jolla, CA, USA) was performed for statistical analysis. Data were represented as mean ± standard deviation (SD). All experiments were repeated at least in three times. The comparisons among multiple groups were made with one-way analysis of variance (ANOVA) followed by Tukey’s test. P < 0.05 was accepted as a statistically significant difference.

## Results

### IS enhanced LPS-induced inflammatory response in THP-1-derived macrophages

**Fig. 1:**
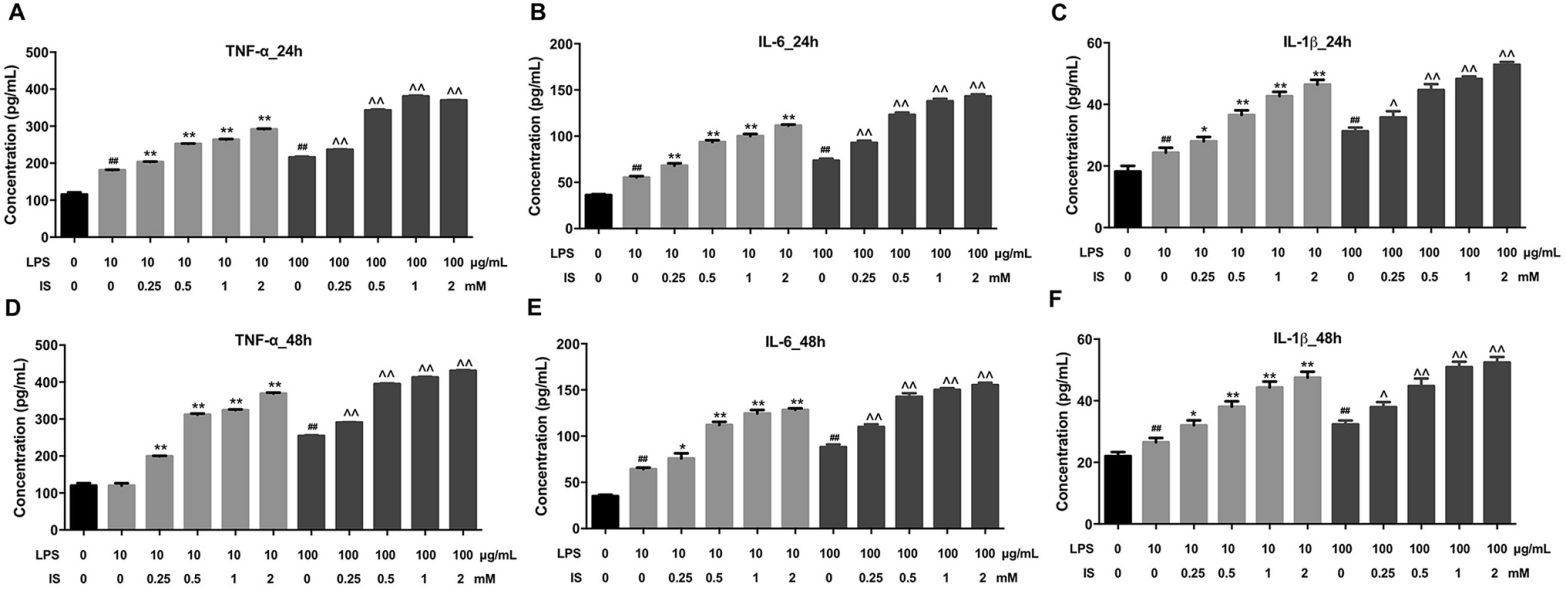
IS enhanced LPS-induced inflammatory response in THP-1-derived macrophages. THP-1 cells were exposed to PMA (160 nM) for 48 h, and then incubated in PMA-free medium for 24 h, following by different concentrations of IS (0, 0.25, 0.5, 1, or 2 mM) and LPS (10 or 100 μg/mL) for 24 h. ELISA assay was used to detect the levels of **a** TNFα, **b** IL-6, **c** IL-1β in macrophages. THP-1-derived macrophages were incubated with different concentrations of IS (0, 0.25, 0.5, 1, or 2 mM) and LPS (10 or 100 μg/mL) for 48 h. ELISA assay was used to detect the levels of **d** TNFα, **e** IL-6, **f** IL-1β in macrophages. ^##^P < 0.01 vs. 0 μg/mL LPS + 0 mM IS group. *P < 0.05, **P < 0.01 vs. 10 μg/mL LPS + 0 mM IS group. ^^^P < 0.05, ^^^^P < 0.01 vs. 100 μg/mL LPS + 0 mM IS group

To investigate the role of IS in inflammatory response in LPS-stimulated macrophages, ELISA assay was applied. LPS significantly induced the production of pro-inflammatory cytokines TNFα, IL-6, IL-1β in THP-1-derived macrophages (Fig. 1a–f). Meanwhile, IS (from 0.25 to 2 mM) markedly increased the production of TNFα, IL-6, IL-1β in LPS-stimulated macrophages when cells were incubated for 24 h compared to macrophages treated with LPS alone (Fig. 1a–f). Similar effects were observed after 48 h of IS treatment (Fig. 1a–f). Therefore, in the following experiments, THP-1-derived macrophages were treated with IS for 24 h. These data indicated that IS could enhance LPS-induced inflammatory response in THP-1 derived macrophages.

### IS promoted M1 macrophage polarization in LPS-stimulated macrophages

**Fig. 2:**
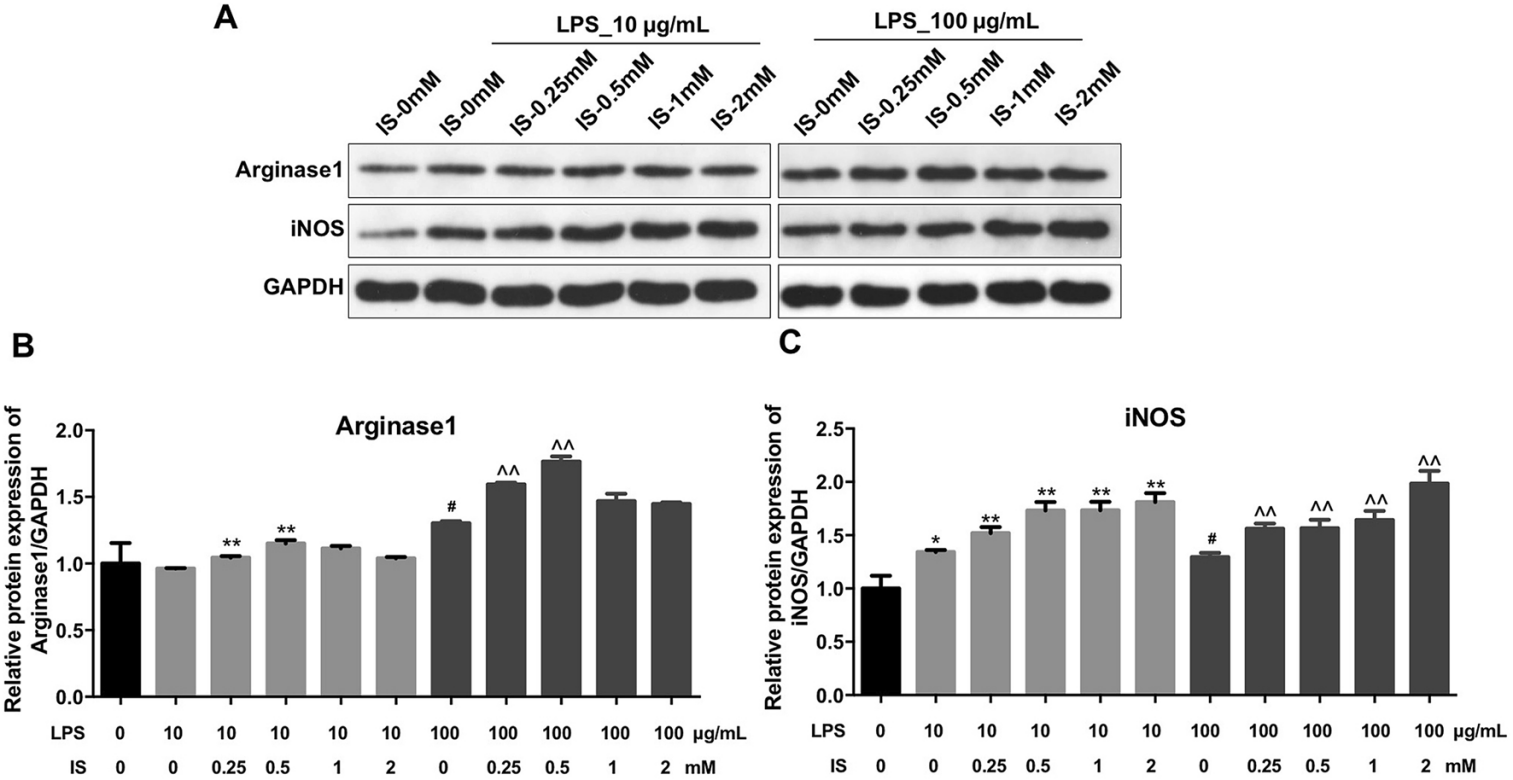
IS upregulated the level of M1 macrophage marker in LPS-stimulated macrophages. **a** THP-1 cells were exposed to PMA (160 nM) for 48 h, and then incubated in PMA-free medium for 24 h, following by different concentrations of IS (0, 0.25, 0.5, 1, or 2 mM) and LPS (10 or 100 μg/mL) for 24 h. Expression levels of Arginase1 and iNOS in macrophages were detected with western blotting. GAPDH was used as an internal control. **b**, **c** The relative expressions of Arginase1 and iNOS in cells were normalized to GAPDH. ^##^P < 0.01 vs. 0 μg/mL LPS + 0 mM IS group. *P < 0.05, **P < 0.01 vs. 10 μg/mL LPS + 0 mM IS group. ^^^P < 0.05, ^^^^P < 0.01 vs. 100 μg/mL LPS + 0 mM IS group

To investigate the effect of IS on macrophage polarization under inflammatory condition, western blot assay was used. As shown in Fig. 2a, b, 2 mM IS had no effect on the expression of M2 macrophage biomarker arginase1 in LPS-stimulated macrophages. Additionally, 2 mM IS notably increased the level of M1 macrophage biomarker iNOS in macrophages in the presence of LPS (10 or 100 μg/mL) (Fig. 2a, c). Meanwhile, no difference in the expression of iNOS were detected between 10 μg/mL LPS alone and 100 μg/mL LPS alone treatment group (Fig. 2a, c). Therefore, 10 μg/mL LPS was utilized in the following experiments.

**Fig. 3:**
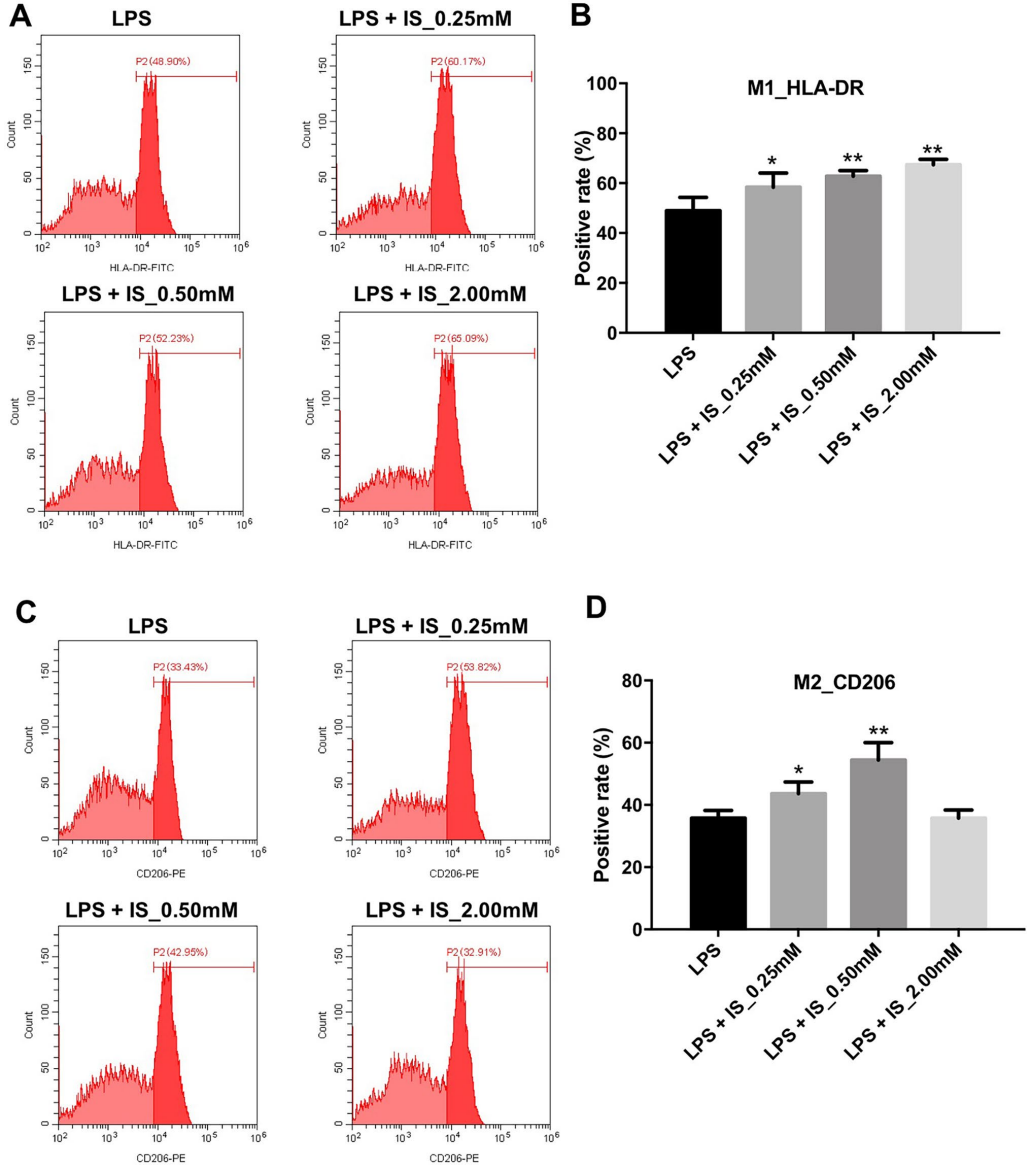
IS promoted M1 macrophage polarization in LPS-stimulated macrophages. THP-1 cells were exposed to PMA (160 nM) for 48 h, and then incubated in PMA-free medium for 24 h, following treated with different concentrations of IS (0, 0.25, 0.5, or 2 mM) and LPS (10 μg/mL) for 24 h. **a**, **b** Representative FACS plots of M1 macrophages (HLA-DR). Percentages of HLA-DR-positive cells were detected by flow cytometry. **c**, **d** Representative FACS plots of M2 macrophages (CD206). Percentages of CD206-positive cells were detected by flow cytometry. *P < 0.05, **P < 0.01 vs. 10 μg/mL LPS group

Next, to further investigate the effect of IS on macrophage polarization in LPS-stimulated macrophages, flow cytometry analysis was used to analyze the proportion of HLA-DR^+^ (M1 macrophage marker) and CD206 (M2 macrophage marker) macrophages. As shown in Fig. 3a, b,  2 mM IS markedly increased the proportion of HLA-DR^+^ cells in LPS-stimulated macrophages compared to macrophages treated with LPS alone; however, 2 mM IS caused no major change in the proportion of CD206^+^ cells during LPS challenge (Fig. 3c, d). These data indicated that IS could promote M1 macrophage polarization in LPS-stimulated macrophages.

### IS inhibited β-catenin signaling in LPS-stimulated macrophages

**Fig. 4:**
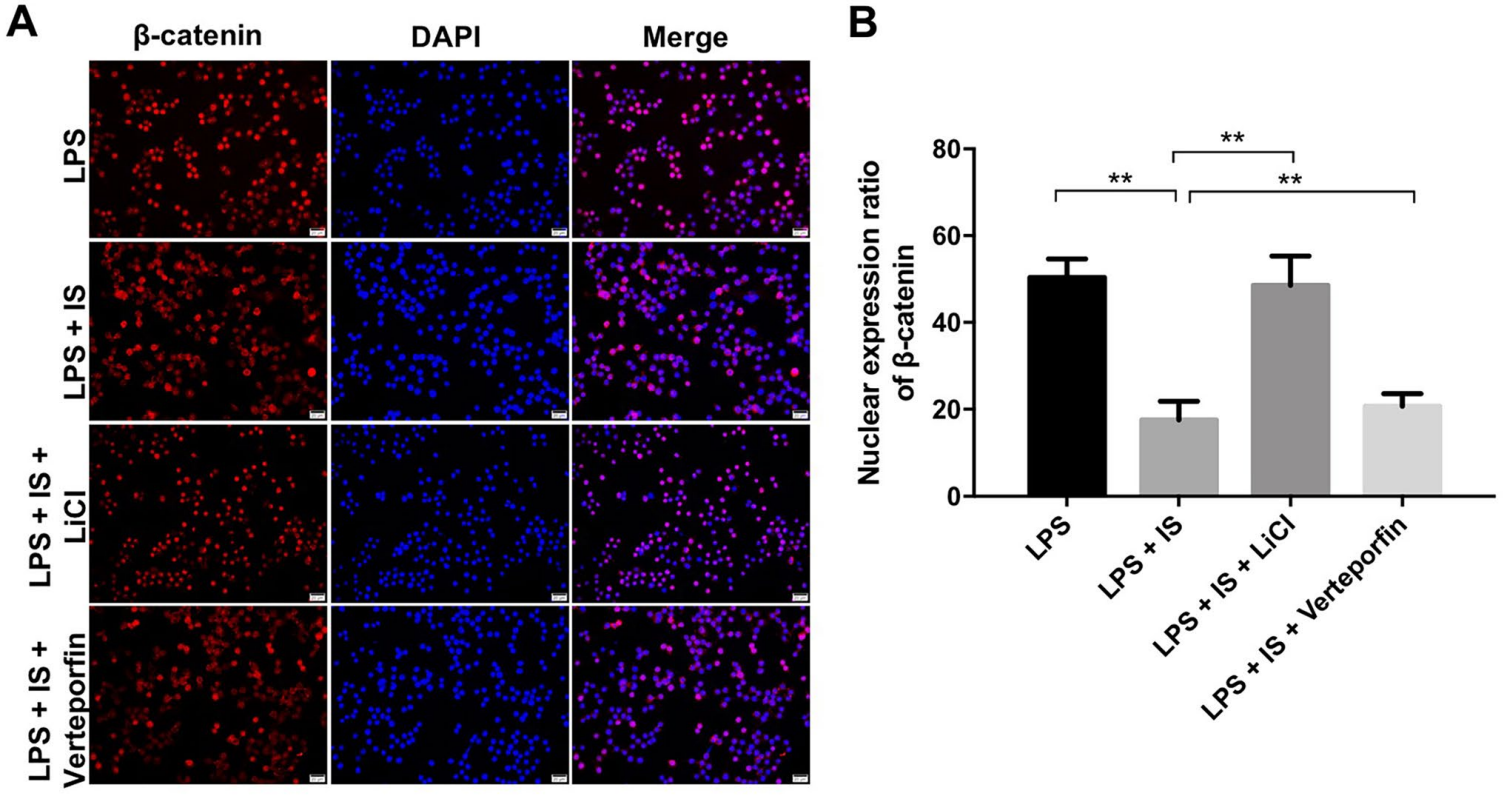
IS inhibited β-catenin signaling in LPS-stimulated macrophages. THP-1 cells were exposed to PMA (160 nM) for 48 h, and then incubated in PMA-free medium for 24 h, following treated with 2 mM IS + 10 μg/mL LPS, plus LiCl (or verteporfin) for 24 h. **a**, **b** Relative fluorescence expression levels were quantified by β-catenin and DAPI staining in macrophages. **P < 0.01

It has been shown that pro-inflammatory cytokines released during inflammation could trigger some molecular signaling cascades, including Wnt/β-catenin signaling (Qu et al. 2018). Feng et al. indicated that activation of Wnt/β-catenin signaling could induce M2 macrophage polarization (Feng et al. 2018). In order to investigate whether IS affects the β-catenin signaling pathway in LPS-stimulated macrophages, immunofluorescence assay was performed. As shown in Fig. 4a, b, IS significantly decreased the nuclear protein level of β-catenin in LPS-stimulated macrophages compared to macrophages treated with LPS alone; however, that effect was reversed by β-catenin signaling activator LiCl or YAP signaling inhibitor verteporfin (Fig. 4a, b). All these results suggested that IS could inhibit β-catenin signaling in LPS-stimulated macrophages.

### IS activated YAP signaling in LPS-stimulated macrophages

**Fig. 5:**
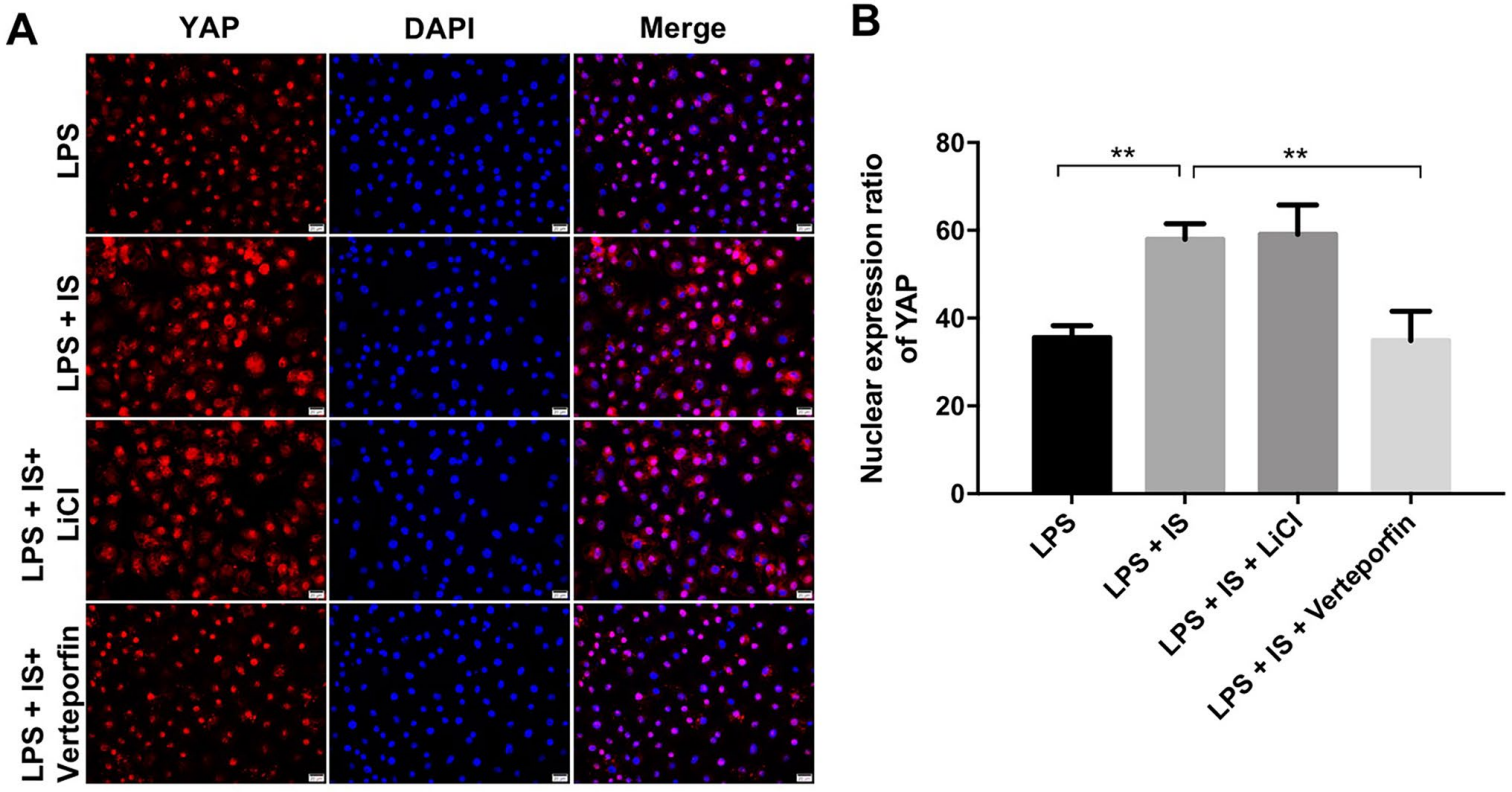
IS activated YAP signaling in LPS-stimulated macrophages. THP-1 cells were exposed to PMA (160 nM) for 48 h, and then incubated in PMA-free medium for 24 h, following treated with 2 mM IS + 10 μg/mL LPS, plus LiCl (or verteporfin) for 24 h. **a**, **b** Relative fluorescence expression levels were quantified by YAP and DAPI staining in macrophages. **P < 0.01

Evidence has been shown that YAP is a core component of the Hippo pathway, which could promote inflammation response in hepatocytes (Mooring et al. 2019). Zhou et al. indicated that YAP could aggravate inflammatory bowel disease via promoting M1 macrophage polarization (Zhou et al. 2019a). As indicated in Fig. 5a, b, IS obviously increased the nuclear protein level of YAP in LPS-stimulated macrophages; however, this phenomena was reversed by verteporfin. These results indicated that IS could activate YAP signaling in LPS-stimulated macrophages.

### IS enhanced inflammatory response in LPS-stimulated macrophages via regulating β-catenin and YAP signaling pathways

**Fig. 6:**
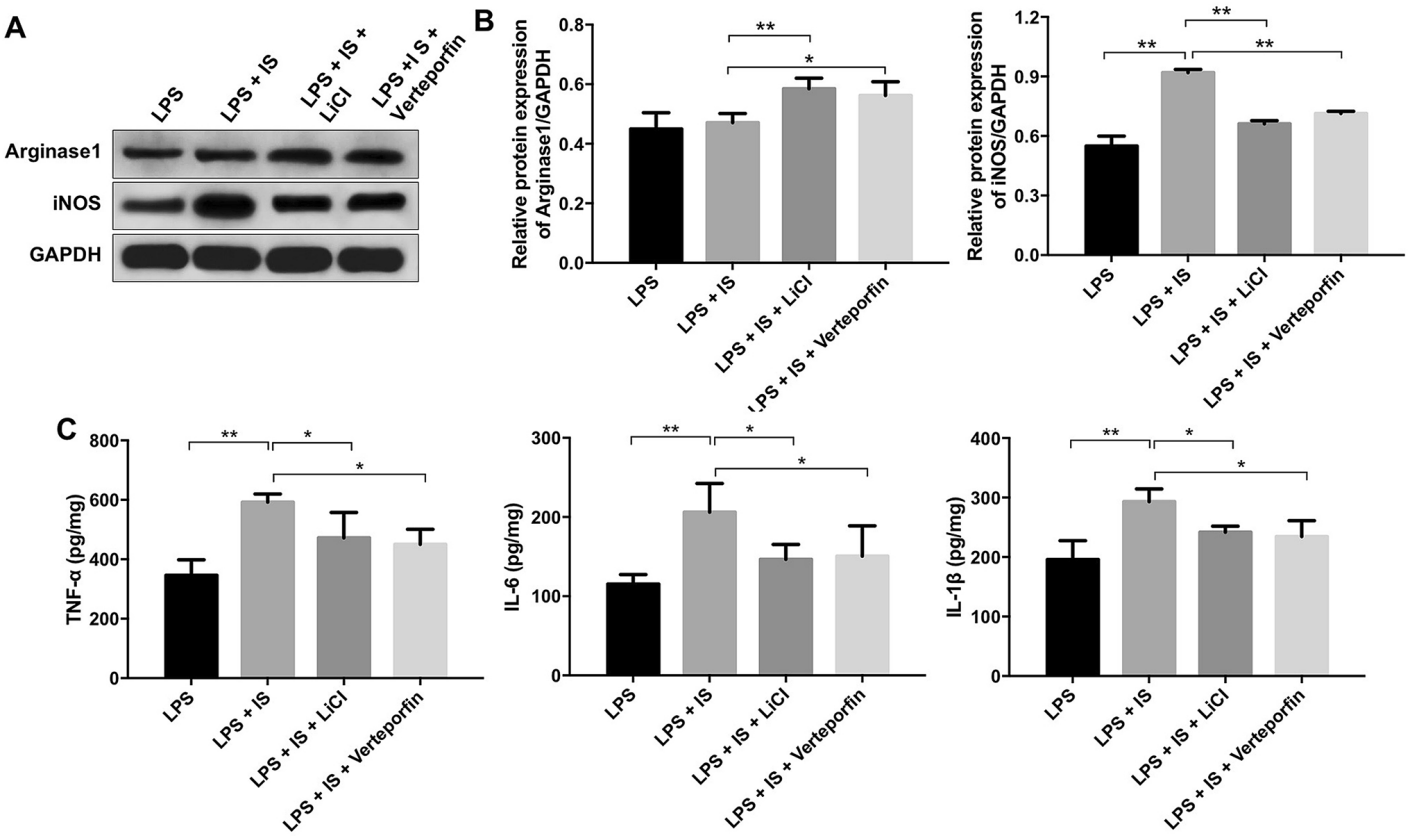
IS enhanced inflammatory response in LPS-stimulated macrophages via regulating β-catenin and YAP signaling pathway. THP-1 cells were exposed to PMA (160 nM) for 48 h, and then incubated in PMA-free medium for 24 h, following treated with 2 mM IS + 10 μg/mL LPS, plus LiCl (or verteporfin) for 24 h. **a** Expression levels of Arginase1 and iNOS in macrophages were detected with western blotting. GAPDH was used as an internal control. **b** The relative expressions of Arginase1 and iNOS in cells were quantified via normalization to GAPDH. **c** ELISA assay was used to detect the levels of TNFα, IL-6, IL-1β in macrophages. **P < 0.01

We next explore whether activation of β-catenin or inhibition of YAP could affect IS-induced inflammatory response in LPS-stimulated macrophages. As shown in Fig. 6a, b, LiCl or verteporfin treatment significantly increased the expression of arginase1 and decreased the level of iNOS in LPS and IS co-treated macrophages. Moreover, IS increased the production of TNFα, IL-6, IL-1β in LPS-stimulated macrophages; however, these effects were markedly reversed by LiCl or verteporfin treatment (Fig. 6c). These data illustrated that activating β-catenin signaling or inhibiting YAP signaling could suppress the IS-induced inflammatory response in LPS-stimulated macrophages by inhibiting M1 macrophage polarization.

## Discussion

Evidences have shown that the concentration of uremic toxin IS in patients are positive corrected with the severity of CKD and CVD (Vanholder et al. 2014; Watanabe et al. 2019). Zhao et al. indicated that IS could be used as a potential biomarker for the diagnosis and treatment of renal fibrosis (Zhao et al. 2016). In addition, IS exhibited a proinflammatory effect in macrophage in CKD, which could function as an indicator of kidney function and a marker of inflammation status (Kaminski et al. 2019; Nakano et al. 2019). In this study, we found that IS could increase the production of TNFα, IL-6, IL-1β in LPS-stimulated macrophages. Adesso et al. indicated that IS markedly increased the production of TNF-α and IL-6 in LPS-stimulated macrophages, which was consistent with our results (Adesso et al. 2013). These data suggested that IS could enhance inflammatory response during LPS challenge.

Macrophages have been recognized as a key factor in the progression of renal fibrosis (Chen et al. 2017). Cao et al. indicated that M1 macrophage is the predominant macrophage phenotype in the early stages of kidney disease, inducing renal inflammation by production proinflammatory mediators (Cao et al. 2013). Meanwhile, macrophage polarization from M1 to M2 was observed in the late stage of kidney disease, and accumulated M2 macrophages promoted kidney fibrosis (Feng et al. 2018). In this study, we found that IS could promote M1 macrophage polarization in LPS-stimulated macrophages. Li et al. indicated that macrophages in CKD displayed enhanced M1 and impaired M2 polarization in response to LPS, which was consistent with our results (Li et al. 2015). Our finding illustrated that IS could enhance inflammatory response LPS-stimulated in macrophages via promoting M1 macrophage polarization.

β-catenin signaling plays an important role in the development of CKD (Li et al. 2017). Activation of β-catenin aggravated kidney dysfunction and promoted renal inflammation (Li et al. 2017). In contrast, manoharan et al. found that activation of the β-catenin pathway could suppress chronic inflammation in DCs (Manoharan et al. 2014). These reports suggested that β-catenin plays a dual role in the regulation of inflammatory response. In this study, we found that β-catenin activator LiCl significantly suppressed the IS-induced inflammatory response in IS-stimulated macrophages, suggesting that β-catenin plays an anti-inflammatory role in IS-induced inflammatory response. In addition, β-catenin activator LiCl markedly upregulated the level of Arginase1, and downregulated the level of iNOS in IS-stimulated macrophages, indicating that β-catenin could promote M2 macrophage polarization but inhibit M1 macrophage polarization. Feng et al. indicated that activation of β-catenin signaling promotes kidney fibrosis through promoting M2 macrophage polarization, which was consistent with our results (Feng et al. 2018). These data indicated that IS could promote M1 macrophage polarization via downregulating β-catenin signaling. Meanwhile, activating β-catenin signaling could suppress the IS-induced inflammatory response in LPS-stimulated macrophages by inhibiting M1 polarization.

YAP is a key transcription coactivator of the Hippo pathway (Murakami et al. 2017). Previous study indicated that YAP is related to inflammation-related diseases (Murakami et al. 2017). Zhou et al. found that YAP could aggravate inflammatory bowel disease, indicating that YAP could inhibit M2 macrophage polarization, and promote M1 macrophage polarization (Zhou et al. 2019a). In this study, IS significantly increased the level of YAP in LPS-stimulated macrophages. In addition, verteporfin markedly decreased the expression of iNOS in IS-stimulated macrophages, indicating that YAP downregulation could inhibit M1 macrophage polarization. Moreover, downregulation of YAP alleviated IS-induced inflammatory response in LPS-stimulated macrophages. These data indicated that IS could promote M1 macrophage polarization via upregulating YAP signaling. Meanwhile, inhibiting YAP signaling could inhibit the IS-induced inflammatory response in LPS-stimulated macrophages by inhibiting M1 polarization.

## Conclusion

In this study, the results indicated that IS could enhance inflammatory response in LPS-stimulated macrophages. Meanwhile, IS could induce M1 macrophage polarization in LPS-stimulated macrophages via inhibiting β-catenin signaling and activating YAP signaling. This study provided evidences that activation of β-catenin or inhibition of YAP could alleviate IS-induced inflammatory response in LPS-stimulated macrophages. This finding may contribute to the understanding of immune dysfunction observed in chronic kidney disease and cardiovascular disease.
